# Disruption of the *Zdhhc9* intellectual disability gene leads to behavioural abnormalities in a mouse model

**DOI:** 10.1016/j.expneurol.2018.06.014

**Published:** 2018-10

**Authors:** Marianna Kouskou, David M. Thomson, Ros R. Brett, Lee Wheeler, Rothwelle J. Tate, Judith A. Pratt, Luke H. Chamberlain

**Affiliations:** Strathclyde Institute of Pharmacy and Biomedical Sciences, University of Strathclyde, Glasgow G4 0RE, United Kingdom

**Keywords:** zDHHC enzymes, zDHHC9, Intellectual disability, H-Ras, S-acylation, Palmitoylation

## Abstract

Protein S-acylation is a widespread post-translational modification that regulates the trafficking and function of a diverse array of proteins. This modification is catalysed by a family of twenty-three zDHHC enzymes that exhibit both specific and overlapping substrate interactions. Mutations in the gene encoding zDHHC9 cause mild-to-moderate intellectual disability, seizures, speech and language impairment, hypoplasia of the corpus callosum and reduced volume of sub-cortical structures. In this study, we have undertaken behavioural phenotyping, magnetic resonance imaging (MRI) and isolation of S-acylated proteins to investigate the effect of disruption of the *Zdhhc9* gene in mice in a C57BL/6 genetic background. *Zdhhc9* mutant male mice exhibit a range of abnormalities compared with their wild-type littermates: altered behaviour in the open-field test, elevated plus maze and acoustic startle test that is consistent with a reduced anxiety level; a reduced hang time in the hanging wire test that suggests underlying hypotonia but which may also be linked to reduced anxiety; deficits in the Morris water maze test of hippocampal-dependent spatial learning and memory; and a 36% reduction in corpus callosum volume revealed by MRI. Surprisingly, membrane association and S-acylation of H-Ras was not disrupted in either whole brain or hippocampus of *Zdhhc9* mutant mice, suggesting that other substrates of this enzyme are linked to the observed changes. Overall, this study highlights a key role for zDHHC9 in brain development and behaviour, and supports the utility of the *Zdhhc9* mutant mouse line to investigate molecular and cellular changes linked to intellectual disability and other deficits in the human population.

## Highlights

• *Zdhhc9* mutant mice display hypotonia and reduced anxiety.
• *Zdhhc9* mutant mice have an altered performance in the Morris water maze.
• *Zdhhc9* mutant mice have reduced corpus callosum volume.
• Membrane binding and S-acylation of H-Ras is not affected in *Zdhhc9* mutant brain.

## 1 Introduction

Intellectual disability (ID, formerly known as mental retardation) is a generalised neurodevelopmental disorder occurring in 1–3% of the general population (Maulik et al., 2011). The disorder is characterised by deficits in intellectual functions such as learning and problem solving, and in adaptive functions such as practical and social skills. Genetic factors can cause ID, however, it can also be acquired before or after birth because of infections, environmental factors or brain trauma (Moeschler and Shevell, 2014).

Many genetic factors that cause ID are X-linked and mutations in over 100 genes present on the X chromosome have been shown to cause ID, with males more heavily affected than females (Branchi et al., 2003). Mutations in the *ZDHHC9* gene, located on the X chromosome, lead to mild-to-moderate ID (Raymond et al., 2007), and are associated with seizures sharing features with Rolandic Epilepsy, speech and language problems and deficits in inhibitory control of attention (Baker et al., 2015). Furthermore, these mutations also cause hypoplasia of the corpus callosum, volume reductions in sub-cortical areas such as the thalamus and striatum, and thinning of the cortex (Masurel-Paulet et al., 2014; Baker et al., 2015; Bathelt et al., 2016). The *ZDHHC9* gene encodes an enzyme that belongs to the zDHHC family of S-acyltransferases. These enzymes catalyse protein S-acylation, a reversible post-translational modification involving the attachment of long chain fatty acids such as palmitic acid to cysteine residues (Chamberlain and Shipston, 2015). There are twenty-three zDHHC enzymes in humans that localise predominantly to intracellular compartments, in particular the endoplasmic reticulum and Golgi; zDHHC9 is localised to the Golgi in both neurons and non-neuronal cells and transfected zDHHC9 also enters neurites (Swarthout et al., 2005; Levy et al., 2011). The enzyme is present in a variety of tissues including brain where it is highly expressed, at least at the mRNA level (Swarthout et al., 2005).

Protein S-acylation exerts a range of effects on modified proteins, such as increasing the affinity of soluble proteins for membranes, and regulating the intracellular trafficking and stability of both soluble and transmembrane protein substrates. S-acylation takes place at the cytoplasmic surface of membranes where the active site of the polytopic zDHHC enzymes is positioned (Rana et al., 2018). Previous work has identified H-Ras and N-Ras as possible targets of zDHHC9 (Swarthout et al., 2005). In support of this, zDHHC9 was shown to be a target of miR-134 in somatostatin interneurons (Chai et al., 2013) and depletion of zDHHC9 was suggested to perturb membrane binding of H-Ras, implying zDHHC9-H-Ras is a functional enzyme-substrate pair. In contrast, other work has questioned the specific requirement of zDHHC9 for Ras S-acylation (Rocks et al., 2010).

The mutations identified in *ZDHHC9* create either truncated proteins that lack the catalytic domain of the enzyme or amino acid substitutions in the catalytic domain that decrease the steady state level of enzyme autoacylation, which is intimately linked with S-acyltransferase activity (Raymond et al., 2007; Mitchell et al., 2010, Mitchell et al., 2014; Jennings and Linder, 2012). Therefore, it is likely that deficits caused by these mutations in the *ZDHHC9* gene arise due to a loss of S-acylation of specific substrate proteins.

Despite *ZDHHC9* mutations being associated with ID, to-date there has not been any detailed examination of the effects of *Zdhhc9* mutations on brain and behaviour in genetic models. In this study, we report the behavioural characterization of a *Zdhhc9* mutant mouse line, which uncovered several deficits broadly consistent with phenotypes reported for other mouse models with mutations in ID genes (Peier et al., 2000; Altafaj et al., 2001; Nielsen et al., 2002; Zang et al., 2009). Furthermore, the *Zdhhc9* mutant mice show corpus callosum changes similar to that observed in humans with *ZDHHC9* mutations (Baker et al., 2015). Overall, these findings suggest that this *Zdhhc9* mutant mouse line may provide an excellent model system to dissect the underlying neurodevelopmental changes that lead to ID and other impairments in humans with disruptive *ZDHHC9* mutations.

## 2 Materials and methods

All animal procedures were conducted in the Biological Procedures Unit at the University of Strathclyde in accordance with Home Office procedures (under a personal, a project and an establishment licence for animal work). Mice were housed in pairs in a temperature and humidity controlled room (21 °C, 45–65% humidity) with a 12-h light/dark cycle (lights on at 08:00). Mice had *ad libitum* access to water and food. Testing was carried out daily between 09:00 and 17:00 and in accordance with the Animals (Scientific Procedures) Act 1986 and EU directive 2010/63/EU for animal experiments. The work also observed ARRIVE guidelines.

### 2.1 *Zdhhc9* mutant mice

*Zdhhc9* knockout mice were purchased from the Mutant Mouse Regional Resource Centers (MMRRC, USA). The knockout (KO) strategy employed resulted in the disruption of the first coding exon of the *Zdhhc9* gene, whereby a genomic region of 207 bp including most of the first coding exon (exon 2) of *Zdhhc9* was deleted. The selection cassette that was used for the KO strategy contained both Neomycin (Neo) and LacZ and is 5288 bp long, according to the sequence in the genotyping protocol given by Lexicon Genetics Incorporated, who produced the mutant line (http://mmrrc.mousebiology.org). All mice used in this project were back-crossed onto the C57BL/6 strain at least six times. To generate mice for use in experiments, *Zdhhc9*^+/−^ females were caged with C57BL/6 wild-type males in order to give birth to mutant and wild-type (WT) male mice which were used as experimental mice for behavioural testing when they reached adulthood (8–10 weeks old). All experiments reported herein compared WT and mutant male littermates. Genotyping was performed by Transnetyx.

### 2.2 Nucleic acid extraction from mouse brain tissue

DNA extraction from mouse brain tissue was conducted using the Isolate Genomic DNA Mini kit (Bioline) according to the instructions of the supplier. An RNeasy Lipid Tissue Mini Kit (Qiagen) was used for RNA extraction according to the instructions of the supplier. The concentration and purity of isolated DNA and RNA were measured using a Nanodrop 2000c spectrophotometer (Thermo Scientific).

### 2.3 End-point and quantitative polymerase chain reaction

Oligo d(T)-primed reverse transcription of RNA was conducted using the Tetro cDNA synthesis kit (Bioline). End-point polymerase chain reaction (PCR) was used with KOD polymerase to amplify specific *Zdhhc9* DNA and cDNA sequences. The sequences for all the primers used in this study are shown in the following Table 1. All of the primers were synthesized and supplied by Sigma. Quantitative PCR (RT-qPCR) was performed in an Applied Biosystems StepOnePlus Real-Time PCR System, using the SYBR Select Master Mix (Life Technologies). The PCR reaction consisted of an initial polymerase activation step of 2 min at 95 °C, followed by 40 cycles of a denaturing stage of 15 s at 95 °C and an annealing and extension step at 60 °C for 1 min. A post-PCR melt curve analysis was included for qualitative analyses of the PCR product. Gene expression fold changes were determined using the 2^(−ΔΔCt)^ method (Livak and Schmittgen, 2001) from the RT-qPCR cycle threshold (Ct) values obtained.

**Table 1:** DNA sequences for all the primers used in this study (shown in 5′ to 3′ orientation).

Primer name	Primer Sequence (5′–3′)
Primers for end-point PCR and RT-qPCR	
MA1 F	AAAGCCCATCTTGGACCAGGAAC
MA1 R	TCAACAGCGTGGCCATGGAG
MA2 F	GTGATGGCCGCGTCATGATG
MA2 R	AAGAGCATAGCGGCAAACACAGG
z9v1 F	ATCGTCTATGTGGCCCTCAAATCC
z9v1 R	GGAATGTGTGAAATCCAGTCAGCC
z9v2 F	ATCGTCTATGTGGCCCTCAAATCC
z9v2 R	AGACGGCTTCACACGGACGAAC
Hprt1 F	CTCATGGACTGATTATGGACAGGAC
Hprt1 R	GCAGGTCAGCAAAGAACTTATAGCC
Tbp F	CCGTGAATCTTGGCTGTAAACTTG
Tbp R	GTTGTCCGTGGCTCTCTTATTCTC
MA0 F	TCAGGGAGAAGTCGCTACCACC
3UTR R	TGGCATCTTCTGCCACTGTCTTAA

### 2.4 Hanging wire task

The day following a physical gross examination, each animal was first weighed and then tested in the hanging wire test in order to assess grip strength. The animal was placed on a cage lid that was then given a small shake so that the mouse would grab the lid and then the lid was flipped upside down with the animal hanging approximately 25 cm above a safe surface (benchtop). The time that the animal spent hanging was recorded. Mice were given 3 trials of 2 min maximum and the average time was used. The animals were given a 2-min rest between trials.

### 2.5 Rotarod task

Mice were initially placed onto the rotarod (Ugo Basile/Stoelting), which was rotating at 3 rpm and accelerated up to 21 rpm within a period of 5 min. The animals were left for a maximum of 8 min on the rotarod. The animals completed 3 trials with a 10-min rest in between the trials, and the average time spent on the rotarod was used.

### 2.6 Open field test

Each mouse was placed into a 40 cm × 40 cm open-top box for a 15 min habituation period followed by a 30-min test period. The animals were tracked throughout the whole 45 min task using Noldus ethovision tracking package (version 8.5). The main measures produced were locomotor output (distance travelled) and time in inner and outer edges of the box.

### 2.7 Elevated plus maze

The elevated plus maze consists of two closed and two open arms (each arm is 30 cm long) raised above the floor by 80 cm. Each animal was placed on the centre of the maze and left to explore it for a single trial of 10 min. Noldus ethovision tracking package (version 8.5) was used to track the animals during the task. The main measures taken were distance travelled and time spent in open and in closed arms of the maze.

### 2.8 Startle curve

Mice were exposed to a range of volumes from 65 dB (background level of volume) to 120 dB for 20 min and their startle reactivity was measured by displacement of an accelerometer attached to the restrainer. The instrument used was the SR Lab apparatus from San Diego Instruments.

### 2.9 Pre-pulse inhibition

During the task which lasts for 20 min, animals were exposed to 120 dB startle trials which were preceded with a range of pre-pulses above the background level of 65 dB. The pre-pulse levels were 4, 8 and 16 dB above background. The instrument used was the SR Lab apparatus from San Diego Instruments. The percentage of pre-pulse inhibition was calculated based on the following formula:

$$100\times \frac{\text{Startle reactivity}\;\mathrm{at}\;120\mathrm{dB}-\text{Startle reactivity}\;\mathrm{at}\;\text{prepulse level}}{\text{Startle reactivity}\;\mathrm{at}\;120\mathrm{dB}}$$

### 2.10 Morris water maze

For the purposes of the task, a round black IR-translucent Perspex tank 98 cm in diameter stood on an IR lightbox and was filled with tap water and left overnight to equilibrate at room temperature (21 °C). Then, a round 10 cm transparent platform was submerged 1 cm below the water surface of the maze.

During the first day of the task, a habituation period of 60 s took place where the platform was placed at the centre of the round water maze. Each mouse was placed on the platform and was given a small boost to start swimming and explore the maze. If the animal did not return to the platform within the 60 s of the habituation, the researcher placed it on the platform for 5 s. The animal was then removed from the maze and was allowed to dry under a ceramic heat lamp.

Following the habituation period and for 5 subsequent days, 4 acquisition trials of 60 s maximum took place each day using pseudorandomly various release points that were not close to the platform. The platform was placed at a distance of 20 cm from the maze wall towards the centre of the maze for the acquisition trials. If the mouse did not locate the platform within 60 s, it was placed on the platform for 5 s. Mice were always given 10 min rest between trials in order to avoid hypothermia.

On the 6th day, a single probe trial of 60 s took place where the platform was removed and the time the animal spent swimming in the quadrant where the platform was originally located *versus* the opposite quadrant was measured in order to assess reference memory. On the 7th day of the experiment a visual cue test of 60 s maximum duration was conducted to exclude any visual problems, motivation differences or motor dysfunction. Before the visual cue test, all the external cues were covered as a white curtain was placed around the water maze hanging from the wall. The platform was this time positioned 2 cm above the water level and a yellow flag was placed on it to make it easily visible. The animal was put in the water maze near the maze wall and the time the animal spent to reach the platform was noted.

### 2.11 Fast low angle shot magnetic resonance imaging (FLASH MRI) and analysis

In order to perform *ex vivo* MRI scans of mouse brain, transcardial perfusion with 4% formaldehyde was performed, and the brains were subsequently removed. Brains were stored at 4 °C for 7 days in order to then perform T1 weighted imaging and were sent to the University of Liverpool where the MRI scanning was performed in the Centre for Pre-clinical Imaging. The brain was initially incubated in 1.5% MultiHance contrast agent by Bracco Diagnostics containing 5 mM Gadobenate Dimeglumine in 4% Paraformaldehyde in PBS at 4 °C for 4 days prior to imaging. Gadolinium is the main chemical agent contained in MultiHance that reduces the T1 relaxation time (Raymond and Pierre, 2005). For imaging, the brain was placed into a pool of fluorinated oil within a holder before being put into the magnet. A 28 mm resonator coil was used and all images were acquired at room temperature. The acquisition time was 2 h and 3 min in a 9.4 Tesla pre-clinical horizontal bore Bruker magnet and a resolution of 41 μm. Images were preliminarily reconstructed using Paravision 6.0.1. For analysis, manual morphometry was used. Manual image segmentation and structural volumetric analysis was carried out in Amira™6.01 software. The overall brain, corpus callosum and hippocampus were segmented using a number of semi-automatic segmentation tools within the software. A surface generated module was attached to each of the segmentation labels and a surface view module was then used to obtain a 3D reconstruction. Material statistics were used to evaluate the number of voxels contributing to each of the segmented structures.

### 2.12 Brain homogenisation and tissue fractionation

Diced whole brain or isolated hippocampi were placed in a Dounce homogenizer with Buffer A (25 mM HEPES, 25 mM NaCl, 1 mM EDTA, pH 7.4), and homogenized by 20 strokes and then passed at least 5 times through a syringe with a 26G needle. The brain lysates were then resolved by SDS-PAGE for immunoblotting analysis. For tissue fractionation, the brain lysate was centrifuged at 800 ×*g* for 5 min at 4 °C in order to pellet the nuclei. The supernatant was then centrifuged at 16100 ×*g* for 50 min at 4 °C, and the supernatant (cytosolic fraction) removed and kept for analysis. The pellet containing the membrane fraction was solubilised in buffer A containing 0.5% Triton X-100 (v/v). Equal amounts of cytosol and membrane fractions were resolved by SDS-PAGE for immunoblotting analysis.

### 2.13 Resin-assisted capture of S-acylated proteins (acyl-RAC)

A membrane fraction from brain or hippocampus was treated with MMTS to block free SH groups (100 mM HEPES, 1.0 mM EDTA, 2.5% SDS and 1.25% MMTS) for 1.5 h at 40 °C in a dry bath with frequent vortexing. The sample was then acetone precipitated and the washed pellet resuspended in 400 μl of binding buffer (100 mM HEPES, 1 mM EDTA, 1% SDS). 160 μl of the protein sample was then added to each of two tubes containing activated Thiopropyl sepharose beads (the remaining 80 μl was kept as total input control). Hydroxylamine or Tris (both at a final concentration of 0.5 M) were then added to one tube each, and incubated overnight at room temperature with end-over-end mixing on a benchtop rotator. In order to separate the beads from the unbound proteins, the samples were centrifuged at 800 ×*g* for 5 min at RT. The supernatant was removed and retained as the unbound fraction. The remaining beads were washed 5 times with 1 ml binding buffer, and the proteins subsequently eluted from the beads in Laemmli sample buffer containing 50 mM DTT. The eluted proteins represent the bound fraction. Before subjecting all samples to SDS-PAGE, the final volumes of the bound and unbound fractions were equalised.

### 2.14 Transfection of HEK293T cells

HEK293T cells on poly-d-lysine-coated 24-well plates were transfected with 1 μg of mouse zDHHC constructs (in PEF-BOS-HA vector) using Lipofectamine 2000 according to manufacturer's instructions (Life Technologies, Paisley UK). Cells were lysed in Laemmli sample buffer containing 25 mM DTT approximately 24 h post-transfection.

### 2.15 Immunoblotting

Primary antibodies used for immunoblotting were as follows: mouse beta actin (Abcam, ab8226, 1:3000), rat HA (Roche, 1:1000), rabbit zDHHC9 (St John's Laboratory, STJ92709, 1:1000), rabbit H-Ras (Santa Cruz, SC-520, 1:300), rabbit GAPDH (Cell Signaling, 14C10, 1:1000), mouse syntaxin (Sigma, HPC-1, 1:1000). IRdye-conjugated secondary antibodies were from LICOR (used at 1:10,000). Images were obtained using a LICOR Odyssey infrared scanner.

### 2.16 Statistical analysis

Statistical analysis was conducted using either SPSS version 22 or Minitab version 17 software packages. For the analysis of the RT-qPCR, an unpaired *t*-test in Minitab was used to determine if there was a genotype effect on the normalised ΔCt values of the target genes. For the analysis of hanging wire and rotarod experiments, an unpaired *t*-test in Minitab was used to determine if there was a genotype effect on average times the animals spent on the wire or the rotarod, respectively. For the analysis of the open field test, a general linear model repeated measures (ANOVA) was used in SPSS to determine if there was a genotype or time effect on time spent in zones or velocity of the animals. For the analysis of the elevated plus maze, unpaired *t*-tests were conducted in Minitab to determine if there was a genotype effect on time spent in open/closed arms or distance travelled. For the analysis of the startle curve and pre-pulse inhibition, general linear model repeated measures (ANOVA) was used in SPSS to determine if there was a genotype effect on the startle reactivity or the percentage of the pre-pulse inhibition of the animals. For the analysis of the Morris water maze, a general linear model repeated measures (ANOVA) was used in SPSS to determine if there was a genotype or day effect on the distance travelled, time and velocity of the animals for the first 5 days of the experiment while for the probe trial, the same statistical test was used to determine if there was a genotype or quadrant effect on the time spent in different quadrants. For the visual cue trial of the Morris water maze, an unpaired *t*-test in Minitab was used to determine if there was a genotype effect on time to complete the trial. As or the analysis of the volumetric results of MRI, an unpaired *t*-test in Minitab was used to determine if there is a genotype effect on the volume of hippocampus or corpus callosum. Finally, unpaired *t*-tests in Minitab were conducted to determine if there was a genotype effect on the level of membrane association and S-acylation of H-Ras.

## 3 Results

### 3.1 Initial characterization of Zdhhc9 mutant mice

*Zdhhc9* knockout mice were obtained from MMRRC and breeding was undertaken by crossing female *Zdhhc9*^+/−^ mice with wild-type C57BL/6 males. Because the *Zdhhc9* gene is located on the X chromosome, this breeding strategy was predicted to produce equal numbers of wild-type (WT) and mutant male mice. Back-crossing was undertaken and all results reported herein were from offspring born to parents that had been backcrossed onto a C57BL/6 genetic background to at least the sixth generation. Interestingly, WT and mutant male mice were not born at the expected Mendelian frequency (ratio of 0.35 ± 0.08 for mutant:WT). In contrast, female WT and heterozygous mutant mice were born at the expected frequency (0.96 ± 0.15 for mutant:WT).

**Fig. 1:**
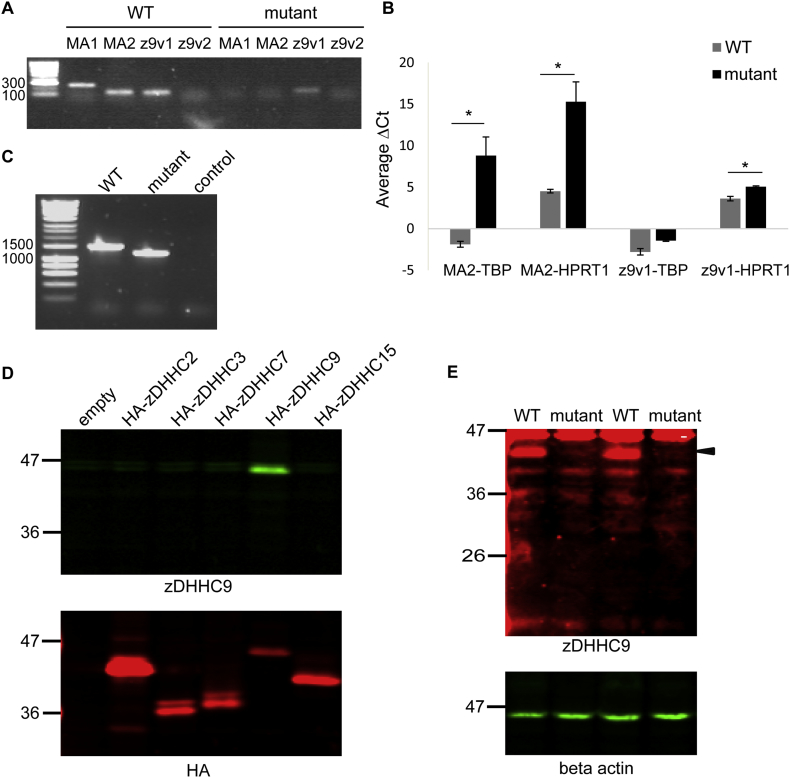
PCR and immunoblotting analysis of *Zdhhc9* expression in wild-type and mutant mice. (A) Agarose gel electrophoresis of end-point PCR products from WT and mutant brain cDNA samples. mRNA confirmation assay 1 (MA1) primers were designed to amplify a *Zdhhc9* mRNA region of 283 bp from the 5′UTR to Exon 2. mRNA confirmation assay 2 (MA2) primers were designed to amplify a *Zdhhc9* mRNA region of 154 bp from Exon 2 to Exon 3. z9v1 primers were designed to amplify a mRNA region of 139 bp from exon 5 to exon 7, and z9v2 primers were designed to amplify a mRNA region of 106 bp from exon 5 to exon 7. HyperLadder 100 bp ladder (Bioline) was used as a marker of DNA size. (B) Comparison of the average ΔCt values of WT and mutant mouse brain samples (*n* = 3 WT, 3 mutant) for MA2 and z9v1 after normalisation against *Tbp* and *Hprt1* reference genes. cDNA from WT and mutant mouse brain was amplified for 40 cycles using specific primers for the different targets (MA2, z9v1, *Tbp* and *Hprt1*) and SYBR Select Master Mix. Statistical analysis (unpaired *t*-test, Minitab version 17) revealed a significant effect of genotype for MA2-*Tbp* (*p* = .042), MA2-*Hprt1* (*p* = .046) and z9v1-*Hprt1* (*p* = .037). *p* value for z9v1-*Tbp* was 0.075. (C) PCR products amplified from cDNA derived from mRNA extracted from WT and *Zdhhc9* mutant mouse brain. Primers were designed to anneal to a region in exon 1 (see MAO F in Table 1 for sequence of primer) and the 3′-UTR (3UTR R in Table 1 for sequence of primer). HyperLadder 1 kb ladder (Bioline) was included as marker of DNA size. (D) Lysates from HEK293T cells transfected with HA-tagged zDHHCs were probed with antibodies against zDHHC9 (*top*) and HA (*bottom*). (E) Brain lysates from WT and mutant mice were probed with antibodies against zDHHC9 (*top*) and beta actin (*bottom*). Position of molecular weight markers is shown on the left of all immunoblots.

Initially, we used end-point and real-time quantitative PCR to investigate *Zdhhc9* expression in WT and *Zdhhc9* mutant mouse brain. Oligonucleotide primers (MA1) were synthesized for the amplification of a cDNA region from the 5’ UTR to exon 2 (exon 2 is disrupted in the *Zdhhc9* mutant mouse line). In addition, an alternative set of primers (MA2) were designed for the amplification of a fragment between exons 2 and 3. As expected, these primers did not give any detectable signal from mutant samples whereas bands of the expected size were amplified from WT samples in endpoint PCR (Fig. 1A). Two other sets of primers were designed to amplify a fragment between exon 5 and exon 7 (z9v1 and z9v2). The purpose of these primers was to test if any mRNA species were generated from the mutant mice downstream of the targeted exon. Z9v2 primers did not support successful PCR amplification from either WT or mutant tissue, however z9v1 primers amplified a band of the expected size from both samples, albeit the band intensity was weaker in the mutant than WT (Fig. 1A). Quantitative real-time PCR (RT-qPCR) was then performed using MA2 and z9v1 primer sets and ΔCt values calculated by comparison with two different reference genes: hypoxanthine guanine phosphoribosyl transferase (*Hprt1*) and TATA box binding protein (*Tbp*) (Fig. 1B). Calculation of ΔΔCt values (mutant-WT) allowed the effective fold-change for MA2 and z9v1 in mutant *versus* WT samples to be determined as: (a) 1639-fold decreased expression (normalised against *Tbp*) and 1754-fold decreased expression (normalised against *Hprt1*) for MA2 in mutant samples; and (b) z9v1 was present in mutant samples at 39.2% and 36.9% of WT levels when normalised against *Tbp* and *Hprt1*, respectively. Statistical analysis (unpaired *t*-test, Minitab version 17) revealed a significant effect of genotype for MA2-Tbp (p = .042), MA2-Hprt1 (p = .046) and z9v1-Hprt1 (p = .037). The p value for z9v1-Tbp was 0.075.

To further investigate the *Zdhhc9* transcript that was amplified by z9v1 primers from mutant samples, primers complementary to the untranslated exon 1 and the 3′-UTR of *Zdhhc9* were designed. These primers amplified a shorter transcript from mutant samples than WT samples (Fig. 1C). Sequencing of the PCR products revealed that the mutant transcript was 290 nucleotides shorter than the WT transcript and lacked the normal ATG initiation codon of zDHHC9. Thus, the mutant mice lack mRNA encoding full-length zDHHC9. However, translation could initiate from a different position in the truncated mutant mRNA fragment; indeed, there is an in-frame ATG present at a more distal position of the sequence that if used would result in a truncated protein lacking all of TMD1 and part of TMD2 but with an intact DHHC-CR domain. To investigate protein expression in the mutant mouse line, we used a commercial antibody generated against a C-terminal peptide of zDHHC9. This peptide is present in the putative truncated protein and thus we could investigate if a shorter protein fragment is indeed formed in the mutant mouse line. The commercial antibody showed good specificity for zDHHC9 and did not cross-react with four other zDHHC isoforms that were tested (Fig. 1D). Analysis of whole brain lysates from WT and mutant mice confirmed loss of full-length zDHHC9 and the absence of any truncated protein fragment in mutant mice (Fig. 1E). Thus, the mutant mice appear to be true knockouts of zDHHC9.

### 3.2 Zdhhc9 mutant mice exhibit behavioural abnormalities

Comparison of *Zdhhc9* mutant mice with their WT littermates revealed no obvious gross abnormalities, however the mutant mice were significantly lighter than WT (25.4 g *versus* 27.6 g, 9 weeks old, *n* = 18 mutant, 24 WT, t(38) = −3.3, p = .002, unpaired *t*-test, Minitab version 17).

**Fig. 2:**
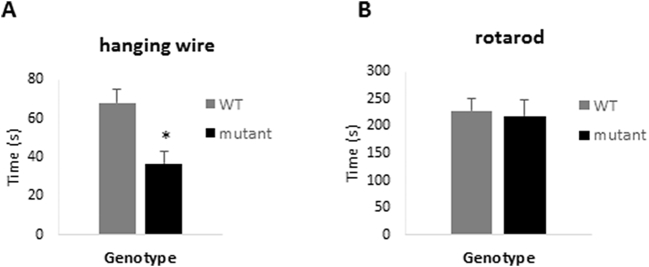
Comparison of wild-type and *Zdhhc9* mutant mice in hanging wire and rotarod tests. (A) Average time spent by mice on the wire. Statistics were conducted using an unpaired *t*-test in Minitab version 17, t(31) = −3.3, *p* = .002 for effect of genotype on time spent on the wire. (B) Average time spent by mice on the rotarod. Statistics were conducted using an unpaired *t*-test in Minitab version 17, ns for genotype (*n* = 20 WT, 14 mutant).

WT and mutant mice were assessed using a hanging wire test. As can be seen in Fig. 2A, the mutant mice spent a significantly shorter period of time hanging on the wire, which is consistent with hypotonia. (t(31) = −3.3, p = .002). Interestingly, despite this deficit, the mutant mice performed at the same level as WT mice in a rotarod test (Fig. 2B), indicating that there was no underlying change in coordination or balance.

**Fig. 3:**
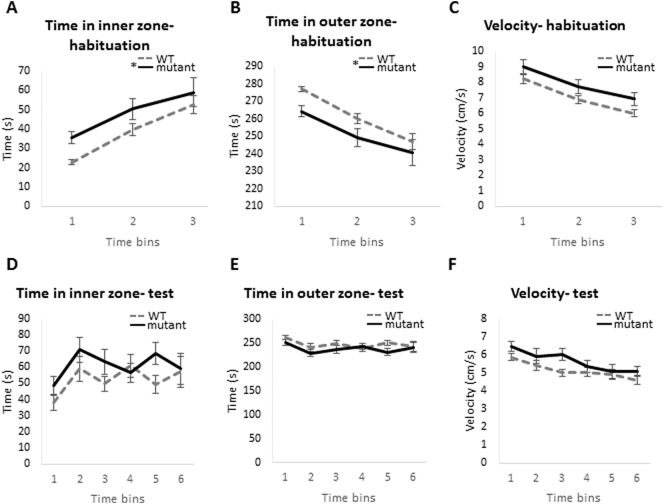
Comparison of wild-type and *Zdhhc9* mutant mice in the open field test. Time spent by WT and mutant mice in the outer and inner sections of the test and their velocity during this time. (A–C) displays these parameters during the habituation phase of the test; (D–F) displays these parameters during the test time (*n* = 14 mutant, 20 WT). Statistics were conducted using general linear model, repeated measures in SPSS version 22 with time bin as the within-subjects factor and genotype as the between-subjects factor; F(1,32) = 4.646, *p* = .039 for effect of genotype in time spent in inner and outer zones and F(1,32) = 3.44, *p* = .073 for effect of genotype on velocity during habituation. Moreover, F(2,64) = 32.573, *p* < .001 for effect of time bin on time spent in inner and outer zones during habituation. There was no interaction between time bin and genotype for the time spent in inner and outer zones, F(2,64) = 0.482, *p* = .482. During test time, p = ns (non-significant) for effect of genotype on time spent in inner and outer zones and velocity. Each time bin represents a period of 5 min.

In order to assess the locomotor activity of the mutant mice, an Open Field Test (OFT) was conducted. Mice were placed individually in the testing arena and their motion was recorded over a period of 15 min (habituation period). This was followed by a 30-min test period. The results are presented in time bins of 5 min. A significant difference was noted in thygmotactic behaviour between mutant and WT mice, specifically during the habituation period (Fig. 3A and B): mutant mice spent significantly more time in the inner zone of the open field and subsequently less time near the walls (F(1,32) = 4.646, p = .039 for effect of genotype and F(2,64) = 32.573, p < .001 for effect of time bin on time spent in inner and outer zones). There was no interaction between time bin and genotype for the time spent in inner and outer zones (F(2,64) = 0.482, p = .482). The velocity of the animals during this period was not significantly different (Fig. 3C; F(1,32) = 3.44, p = .073 for effect of genotype on velocity during habituation). However, during the test period, there was no significant difference between WT and mutant animals in thygmotactic behaviour (Fig. 3D–F), perhaps suggesting that the difference observed in the habituation period is associated with the mutant mice being in a novel environment.

**Fig. 4:**
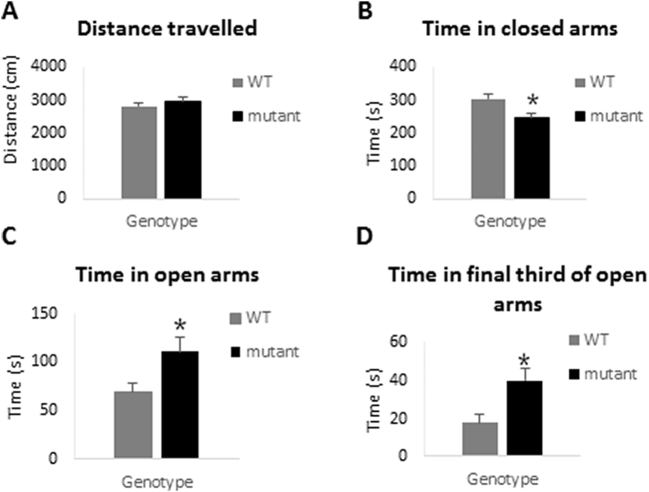
Performance of WT and *Zdhhc9* mutant mice in the elevated plus maze. Distance moved (A), time spent in closed arms (B), time spent in open arms (C), and time spent in final third of open arms (D) are shown (n = 20 WT, 14 mutant). Statistics were conducted using an unpaired *t*-test in Minitab version 17; t(22) = 2.49, *p* = .021 for effect of genotype on time spent in the open arms, t(29) = −2.84, *p* = .008 for effect of genotype on time spent in closed arms and t(24) = 2.74, *p* = .011 for effect of genotype on time spent in the final thirds of open arms.

The thygmotactic behaviour of the *Zdhhc9* mutant mice during the habituation phase of the OFT is consistent with reduced anxiety levels. To examine this more directly, WT and mutant mice were subsequently tested in an Elevated Plus Maze (EPM). The time spent between open and closed arms of this apparatus is used as a measure of anxiety in rodents. Compared to WT mice, *Zdhhc9* mutant mice spent significantly more time exploring the more aversive open arms and final third of the open arms and significantly less time exploring the less aversive closed arms (t(22) = 2.49, p = .021 for effect of genotype on time spent in the open arms, t(29) = −2.84, p = .008 for effect of genotype on time spent in closed arms and t(24) = 2.74, p = .011 for effect of genotype on time spent in the final thirds of open arms) (Fig. 4). Both WT and mutant groups moved a similar distance during the testing period and had similar velocity. These observations fit with the idea that *Zdhhc9* mutant mice do indeed have lower anxiety levels than WT mice.

**Fig. 5:**
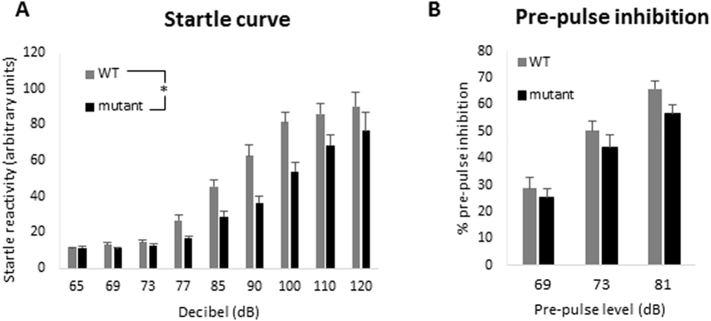
Comparison of acoustic startle and PPI response of wild-type and *Zdhhc9* mutant mice. (A) Startle reactivity of WT and *Zdhhc9* mutant mice. The startle response to a sound at a range of levels (dB) was measured. Statistics were conducted using general linear model, repeated measures in SPSS version 22; F(1,44) = 13.622, *p* = .001 for effect of genotype (*n* = 24 WT, 22 mutant). (B) Mice received a pre-pulse at the levels (dB) shown. Following this, the startle response to a 120 dB sound was measured. Statistics were conducted using general linear model, repeated measures in SPSS version 22; *p* > .05 for effect of genotype (n = 24 WT, 22 mutant).

Consistent with findings of the EPM, mutant mice also displayed reduced acoustic startle reactivity (Fig. 5A) (F(1,44) = 13.622, p = .001 for effect of genotype). The results indicate that the mutant animals begin to exhibit a startle response at higher decibel levels (85 dB and above) compared to WT animals (77 dB). Two days after the startle curve task, the same animals (*n* = 22 mutant, 24 WT) were tested for pre-pulse inhibition to assess sensorimotor gating. This test also provides a sensitive measure of hearing. Animals were exposed to 120 dB startle trials which were preceded with a range of pre-pulses above the background level of 65 dB. The pre-pulse levels were 4, 8 and 16 dB above the background level of 65 dB (*i.e.* 69, 73 and 81 dB). Fig. 5B shows that the mutant mice displayed a similar pattern of pre-pulse inhibition to WT animals, indicating that they have normal sensorimotor gating and hearing ability (F(1,44) = 2.029, p = .161).

**Fig. 6:**
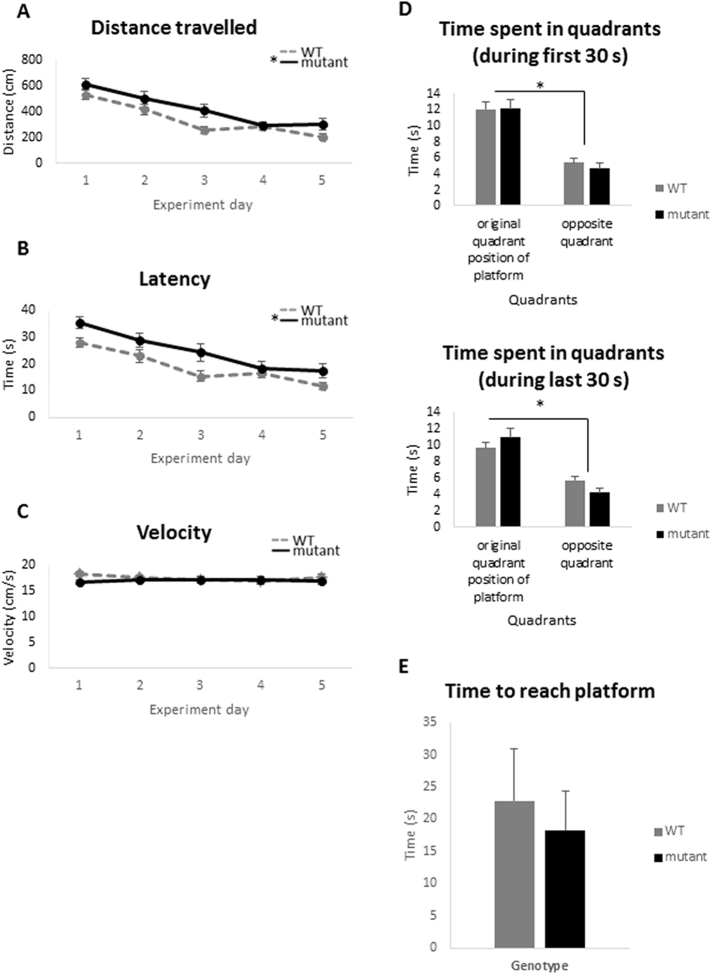
Performance of *Zdhhc9* mutant and WT mice in the Morris water maze. Distance moved (A), latency (B) and mean velocity (C) during the 5 acquisition days of the experiment are shown. Statistics were conducted using general linear model, repeated measures in SPSS version 22 with experiment day as the within-subjects factor and genotype as the between-subjects factor; F(1,44) = 8.935, *p* = .005 for effect of genotype on distance moved, F(4,176) = 27.647, p < .001 for effect of day on distance moved while no interaction was found between day and genotype with F(4,176) = 1.020 and *p* = .399. F(1,44) = 9.677, *p* = .003 for effect of genotype on latency, F(4,176) = 23.499 and p < .001 for effect of day on latency while no interaction was found between day and genotype with F(4,176) = 0.868 and *p* = .484 (*n* = 26 WT, 20 mutant). The effect of genotype on velocity was non-significant. (D) Time spent in quadrants during the first 30 s and last 30 s of the probe trial to assess reference memory are shown. Statistics were conducted using general linear model, repeated measures in SPSS version 22; F(1,44) = 49.573, p < .001 for effect of quadrant during the first 30 s and F(1,44) = 41.805, p < .001 for effect of quadrant during the last 30 s (n = 26 WT, 20 mutant). (E) Time spent to reach the visible platform for WT and Zdhhc9 mutant mice during the visual cue trial of Morris water maze. Statistics were conducted using unpaired *t*-test in Minitab version 17; t(42) = −0.92, p = ns for effect of genotype (n = 26 WT, 20 mutant).

Finally, we also investigated if *Zdhhc9* mutant mice exhibit any deficits in learning and memory by analysing their performance in the Morris water maze (MWM), a task with a strong dependence on hippocampal function (Morris, 1989). The distance travelled and the time spent by WT and *Zdhhc9* mutant mice to find a submerged platform in the water tank was measured over consecutive days for a total of 5 days. Fig. 6 shows that the mutant mice displayed a different pattern of spatial learning with impaired task acquisition. There was a significant effect of genotype on distance moved (F(1,44) = 8.935, p = .005) and as predicted there was a significant effect of day (F(4,176) = 27.647, p < .001). The effect of genotype did not interact with day (F(4,176) = 1.020, p = .399). There was also a significant effect of genotype on latency (F(1,44) = 9.677, p = .003) and for the effect of day on latency (F(4,176) = 23.499, p < .001), although no interaction was found between day and genotype on latency (F(4,176) = 0.868, p = .484 (*n* = 26 WT, 20 mutant)). The effect of genotype on velocity was non-significant (F(1,44) = 0.818, p = .371). The overall effect of genotype on distance moved and latency (duration to complete the task) but not on velocity suggests that the observed deficit is not related to swimming ability. Subsequently, a probe trial took place in order to assess reference memory in the same animals. The platform was removed and the time spent in the quadrant where the platform was *versus* the opposite quadrant was measured. There was a significant effect of quadrant during the first 30 s (F(1,44) = 49.573, p < .001) and during the last 30 s (F(1,44) = 41.805, p < .001) but there was no significant effect of genotype (F(1,44) = 0.057, p = .813). As there was a significant effect of quadrant but not genotype on performance, this indicates that both groups learned where the platform was by the end of the task as they spent significantly more time in the quadrant where the platform was during the 60 s of the probe trial (Fig. 6D). After the probe trial, a visual cue test was conducted on the same animals (20 mutant, 26 WT) in order to exclude the possibility of any motivational or visual confounds. Fig. 6E shows that both WT and mutant mice reached the platform in a similar time indicating that *Zdhhc9* mutant mice did not exhibit proximal visual deficits and were as motivated to complete the task as WT animals (t(42) = −0.92, p = .365).

### 3.3 MRI analysis of WT and Zdhhc9 mutant mouse brains

**Fig. 7:**
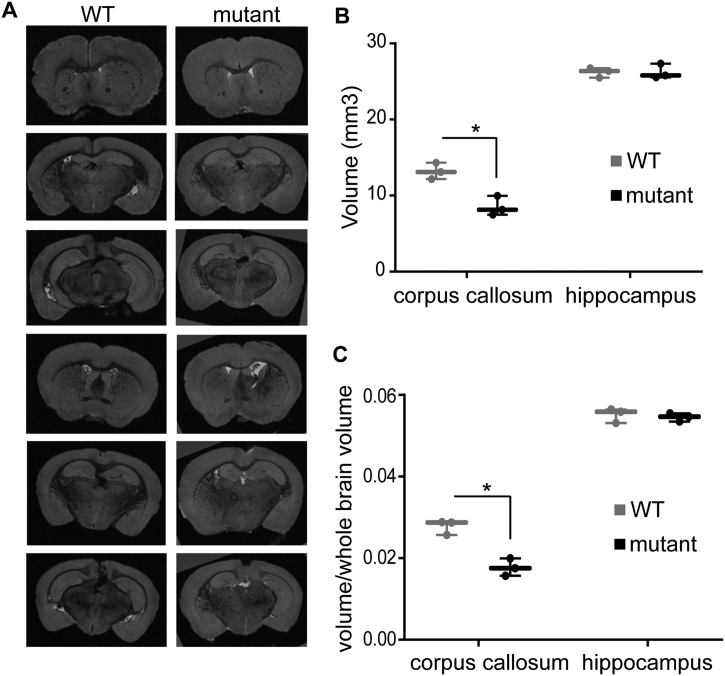
MRI analysis of brains from WT and *Zdhhc9* mutant mice. (A) Coronal images from WT and mutant mouse brains after *ex vivo* MRI scan in a 9.4 Tesla magnet. (B and C) Box and whiskers graphs showing the various data points for the volume of corpus callosum and hippocampus. The whiskers indicate the maximum and minimum data points while the thick horizontal line indicates the median. (B) without normalisation; (C) with normalisation to total brain volume (*n* = 3 WT, 3 mutant).

In order to examine if the *Zdhhc9* mutant mice exhibit shrinkage of corpus callosum, similarly to patients with mutations in this gene (Baker et al., 2015), *ex vivo* MRI scanning of 3 mutant and 3 WT mouse brains was performed. The hippocampus was also analysed because of the importance of this brain region for learning and memory and its role in successful completion of the MWM task in which *Zdhhc9* mutant mice were shown to have a deficit. 3D brain reconstruction (Supplementary Fig. 1) for corpus callosum and hippocampus were completed using Amira software. Coronal, sagittal and transverse plane images (Fig. 7 A and Supplementary Fig. 2) showed possible shrinkage of corpus callosum in mutant mouse brain but no apparent differences were observed in the volume of hippocampus between WT and mutant mice. Volumetric analysis of regions of interest was performed with or without normalisation against the whole brain volume in order to quantify changes in corpus callosum and hippocampal. This analysis showed that the mutant mice had an average 35% reduction in the total volume of corpus callosum but no significant change in hippocampus (Fig. 7B). This reduction was statistically significant (average volume of corpus callosum was 8.5 mm^3^ for mutant and 13.2 mm^3^ for WT mice (p = .017)). Analysis with normalisation against the total brain volume of each sample showed similar results as the analysis without normalisation, with *Zdhhc9* mutant mice displaying an average 36% reduction in the volume of corpus callosum (Fig. 7C) but not in hippocampus. This reduction in corpus callosum volume was also statistically significant (p = .008).

### 3.4 Analysis of H-Ras membrane association and S-acylation in Zdhhc9 mutant mice

**Fig. 8:**
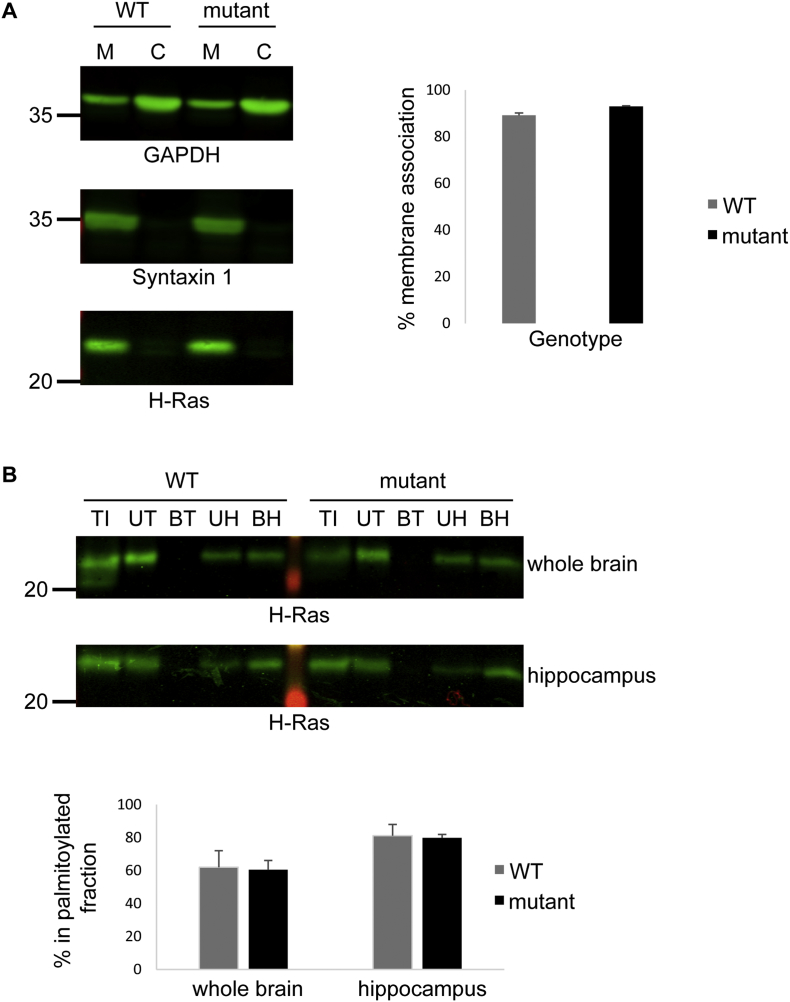
Analysis of membrane association and S-acylation of H-Ras in whole brain and hippocampus from WT and *Zdhhc9* mutant mice. (A) Membrane and cytosol fractions from whole brain homogenates were resolved by SDS-PAGE and transferred to nitrocellulose, and subsequently probed with GAPDH, Syntaxin and H-Ras antibodies. The left panel shows representative immunoblots (position of molecular weight markers is shown on the left), whereas the right panel shows quantified data for % membrane association. Statistical analysis using an unpaired *t*-test indicated that there was no significant difference in H-Ras membrane association between WT and mutant samples; p > .05 (n = 3 WT, 3 mutant). M: membrane fraction, C: cytosolic fraction. (B) Acyl-RAC samples from whole brain or hippocampi were resolved by SDS-PAGE and transferred to nitrocellulose, and subsequently probed with H-Ras antibody. The upper panel shows representative immunoblots (position of molecular weight markers is shown on the left), whereas the lower panel shows quantified data from whole brain or hippocampi. Statistical analysis using an unpaired *t*-test indicated that there was no significant difference in H-Ras S-acylation between WT and mutant samples; p > .05 (n = 3 WT, 3 mutant). TI: total input, UT: Unbound Tris treated fraction, BT: Bound Tris treated fraction, UH: Unbound HA treated fraction, BH: Bound HA treated fraction.

H– and N-Ras have been suggested to be targets of the zDHHC9 enzyme (Swarthout et al., 2005; Chai et al., 2013), and it was suggested that depletion of zDHHC9 leads to a loss of membrane association of H-Ras in somatostatin interneurons. Thus, we investigated if this protein was affected in both whole brain and isolated hippocampi using an antibody that displays specificity towards H-Ras over other Ras isoforms (SC-520) (Waters et al., 2017). Analysis of isolated membrane and cytosolic fractions showed that membrane association of H-Ras was unaffected in *Zdhhc9* mutant mouse brain (Fig. 8A); GAPDH and Syntaxin 1 served as controls for soluble and membrane proteins, respectively. Statistical analysis was conducted using an unpaired *t*-test (p > .05 for effect of genotype on percentage of membrane association of H-Ras). As membrane binding of H-Ras is mediated by S-acylation, we subsequently isolated S-acylated proteins from both whole brain and isolated hippocampus using acyl-RAC. This approach involves removing S-acyl chains by hydroxylamine treatment and then capture of the previously S-acylated proteins on a thioreactive matrix. Fig. 8B shows that there was no significant difference in the level of S-acylation of H-Ras between WT and mutant samples (S-acylated proteins are recovered in the HA bound sample (BH)). Statistical analysis was conducted using an unpaired *t*-test (p > .05 for effect of genotype on the level of H-Ras S-acylation).

## 4 Discussion

Knockout of zDHHC9 expression causes clear effects on behaviour and brain anatomy, which are reminiscent of other mouse models of ID (Peier et al., 2000; Altafaj et al., 2001; Nielsen et al., 2002; Zang et al., 2009). Furthermore, the observation of reduced corpus callosum volume in the *Zdhhc9* mutant mice is strikingly similar to the changes seen in humans with *ZDHHC9* mutations (Baker et al., 2015).

The male *Zdhhc9* mutant mice displayed differences to their WT littermates in many behavioural tasks. The results of several tasks were consistent with the mutant mice having decreased anxiety levels. The EPM is a classical test for anxiety and the *Zdhhc9* mutant mice spent significantly longer than WT animals in the more aversive open arms of the EPM, associated with lower levels of anxiety. Consistent with this, the mutant mice also spent longer in the inner zone of the OFT during the habituation phase and exhibited a reduced acoustic startle response. The reduced acoustic startle response is unlikely to be linked to hearing deficits as the mutant mice exhibited no change in PPI compared with wild-type littermates. Reduced anxiety has been reported in other ID mouse models, including *Fmr1* and *Mecp2* mutant mice and Down syndrome mouse models (Escorihuela et al., 1998; Peier et al., 2000; Altafaj et al., 2001; Samaco et al., 2008; Zang et al., 2009).

The acoustic startle reflex is based on the observation that animals flinch immediately following a sudden stimulus such as a loud noise, and this test can be used as a behavioural tool to assess brain mechanisms of sensorimotor integration. The startle response itself is mediated by neurons in the lower brainstem, whereas pre-pulse inhibition is dependent on a more complex forebrain circuitry (Koch, 1999). Children with anxiety disorders show an increased acoustic startle reflex (Bakker et al., 2009). In accordance with that, in rats the amplitude of acoustic startle can be decreased by anxiolytic drugs (Walker and Davis, 1997) showing a link between anxiety and startle reactivity. As the amygdala is important in processing emotional reactions such as anxiety and fear (Davis, 2006), this brain region could possibly be affected by the disruption of *Zdhhc9* and this merits analysis in future studies.

*Fmr1* KO mice show reduced anxiety indicated by reduced thygmotaxis in the OFT and in the light-dark exploration test (Peier et al., 2000), as well as reduced startle reactivity at high decibel levels (120 dB) of white noise (Nielsen et al., 2002). Another mouse model of Fragile X-syndrome, mice that have the I304N mutation in *Fmr1*, show decreased anxiety behaviour in the OFT and decreased acoustic startle response, as well as hyperactivity and higher exploratory behaviour in the OFT (Zang et al., 2009). The reduced anxiety of the mouse model may appear contradictory to the increased anxiety presented by FXS patients but it is important to note that their anxiety disorder is predominately observed in social settings (Hagerman and Hagerman, 2002).

Interestingly, the *Zdhhc9* mutant mice spent a significantly shorter time on the hanging wire, which is consistent with these mice having a hypotonia phenotype. Interestingly, hypotonia is also present in patients with *ZDHHC9* mutations (Raymond et al., 2007; Masurel-Paulet et al., 2014; Han et al., 2017), and also in a patient with loss of *ZDHHC15* expression and severe ID due to a balanced chromosomal translocation (Mansouri et al., 2005). Furthermore, several other relevant human conditions and mouse models thereof also display characteristic hypotonia, including ADHD (Wells et al., 2016), Down's syndrome (Costa et al., 1999; Roizen, 2001; Scorza and Cavalheiro, 2011), Rett syndrome (Heilstedt et al., 2002; Friez et al., 2006; Samaco et al., 2008) and Fragile X syndrome (Vieregge and Froster-lskenius, 1989). However it is also worthwhile considering that the reduced hang time in this task might be linked to lower anxiety levels in mutant mice. Indeed, there was no deficit in rotarod performance, suggesting that motor coordination and balance are unaffected by the *Zdhhc9* mutation. Other ID mouse models have been reported to exhibit deficits in the hanging wire task, including models of Down's syndrome and Rett syndrome (Costa et al., 1999; Altafaj et al., 2001; Samaco et al., 2008). Furthermore, *Ptchd1* KO mice also exhibited a hanging wire deficit but no change in rotarod performance, similar to that seen here for *Zdhhc9* mutant mice (Wells et al., 2016).

The MWM task is a classical test for changes in hippocampal-dependent spatial learning and memory. The learning profile of the *Zdhhc9* mutant mice in this task was significantly different from WT mice. However, it is interesting to note that the “rate” of learning between WT and mutant mice appeared similar and that mutant mice required a longer period of time to locate the platform on all days of the test; indeed a deficit was clearly present from the outset of the MWM test. Thus, although the results are consistent with a hippocampal-related deficit in learning and memory, it is also formally possible that the altered behaviour of the mutant mice in the MWM reflects a deficit in some other parameter other than spatial learning and memory, such as abnormal context-dependent processing. Nevertheless, it is interesting to note that the profile of learning of mutant and WT mice in the MWM task is similar to that reported in a model of Down's syndrome in which mice overexpress Dyrk1A (Altafaj et al., 2001); these mutant mice acquired the task slower than WT and showed significantly longer escape latencies in the first acquisition sessions.

Aberrations in spatial learning are generally attributed to hippocampal defects (Logue et al., 1997). Changes in hippocampus can affect amygdala based on their reciprocal connections (Bast et al., 2001). Since there was no difference between mutant and WT in the visible condition of the MWM task it is likely that the increased latency of mutant mice in the hidden platform condition is not caused by an underlying motivational, motor, or sensory deficit. Hence, the observed impairment appears to be limited to the spatial abilities of the mouse. This could be connected to the increased exploratory behaviour (*e.g.* OFT and EPM) that might be responsible for excessive early search behaviour. Most patients with *ZDHHC9* mutations have mild to moderate rather than severe ID (Baker et al., 2015). If the ID is presented as a mild/moderate impairment then simple learning and memory tasks may not be challenging enough to create a significant difference between mutant and WT mice. Our data show that the mutant mice are not severely impaired in learning and memory tasks, resembling the human phenotype.

The experimental data suggest that disruption of *Zdhhc9* may disturb processes that involve the hippocampus and amygdala. MRI analysis showed that there was no change in the volume of the hippocampus, suggesting that neuronal numbers in this brain region are similar in WT and mutant mice. Interestingly however, this analysis did reveal a significant reduction in corpus callosum volume, strikingly similar to that seen in humans with *ZDHHC9* mutations (Baker et al., 2015). The corpus callosum is the largest white matter tract in the human brain, important for interhemispheric communication (Luders et al., 2010). Volumetric reduction of this area could be explained by reduced white matter in the mutant mouse brain. Decreased white matter volume has also been reported by MRI analysis of *Zdhhc17*-KO mice, and this was not due to changes in astrocyte or microglial cell numbers but to neuronal cell loss (Singaraja et al., 2011). Consistent with our MRI analysis of the hippocampus in *Zdhhc9* mutant mice, hippocampal volume was also unaffected in humans with *ZDHHC9* mutations.

Corpus callosum abnormalities have been described in a variety of syndromes and genetic disorders. Thinning of corpus callosum has also been described as a consistent finding in adolescents with ID (Spencer et al., 2005). Another study concluded that adults with ID show abnormal white matter integrity (Yu et al., 2008). Children with Down Syndrome also have reduced volume of corpus callosum compared to healthy controls (Gunbey et al., 2017). Moreover, hypoplasia of corpus callosum has been described in ADHD and autism (Paul, 2011).

Mutations in *ZDHHC9* that cause ID lead to defects in S-acylation activity, implying that ID most likely arises due to loss of S-acylation of specific substrate protein(s). Previous work has suggested that Ras proteins are likely to be targets of zDHHC9 (Swarthout et al., 2005; Chai et al., 2013). Interestingly mutations in the genes encoding H-Ras and N-Ras are associated with ID (Denayer et al., 2008; Cirstea et al., 2010; Gremer et al., 2010; Lorenz et al., 2013). Using an antibody reported to be specific for H-Ras (https://www.scbt.com/scbt/product/h-ras-antibody-c-20), it was demonstrated that the association of this protein with membranes does not change in *Zdhhc9* mutant mice. This was a surprising observation as stable membrane association of this protein is dependent on S-acylation (Hancock et al., 1989). This finding suggested that S-acylation of H-Ras is not disrupted in *Zdhhc9* mutant mice and this was confirmed by subsequent immunoblotting analysis of isolated Acyl-RAC fractions. However, it is possible that changes in S-acylation of H-Ras could be occurring in specific brain regions and that these effects were diluted out by the analysis of whole brain samples. However as no change in S-acylation was detected in the hippocampus, this suggests that the deficits observed in the hippocampal-dependent MWM task are not linked to changes in Ras S-acylation. Rocks et al. (2010) suggested that knockdown of zDHHC9 did not affect the palmitoylation-dependent intracellular localisation of semi-synthetic N-Ras proteins in HeLa cells, whereas Liu et al. (2016) reported that Ras palmitoylation was reduced but not completely inhibited in bone marrow cells from *Zdhhc9* mutant mice. Thus, palmitoylation of Ras proteins may display a different dependence on zDHHC9 depending on cell type.

Overall, the behavioural phenotypes and changes in corpus callosum volume suggest that *Zdhhc9* knockout mice represent a valuable model with which to investigate the underlying molecular and cellular changes that occur in ID and other neurological impairments caused by *ZDHHC9* mutations.

Declaration of interests: none.

## Appendix A Supplementary data

Supplementary material Image 1
