# Supplementary material for: Disruption of the Zdhhc9 intellectual disability gene leads to behavioural abnormalities in a mouse model
Source: Exp Neurol. 2018 Oct;308:35–46. doi: 10.1016/j.expneurol.2018.06.014 (PMC6104741; doi:10.1016/j.expneurol.2018.06.014)
Supplement: Supplementary file 1 — Supplementary material [file mmc1.pdf]

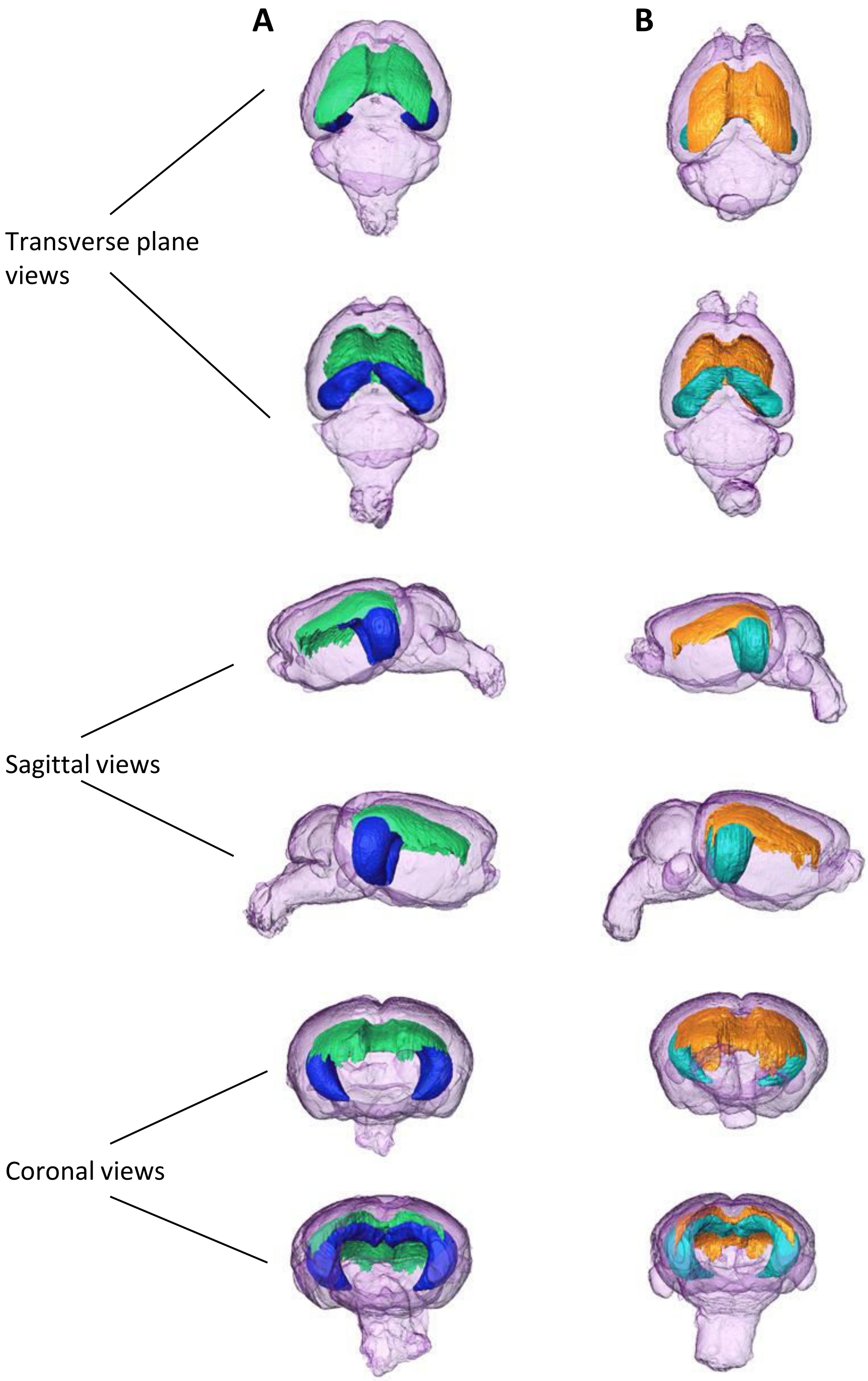

Supplementary Figure 1. Transverse plane, sagittal and coronal images from 3D reconstruction of representative WT (A) and mutant (B) mouse brains using Amira 6.01 software after ex vivo MRI scan. Volumetric analysis was conducted for hippocampus (dark blue in panel A and turquoise in panel B), corpus callosum (green in panel A and orange in panel B) and whole brain (light purple in both panels).

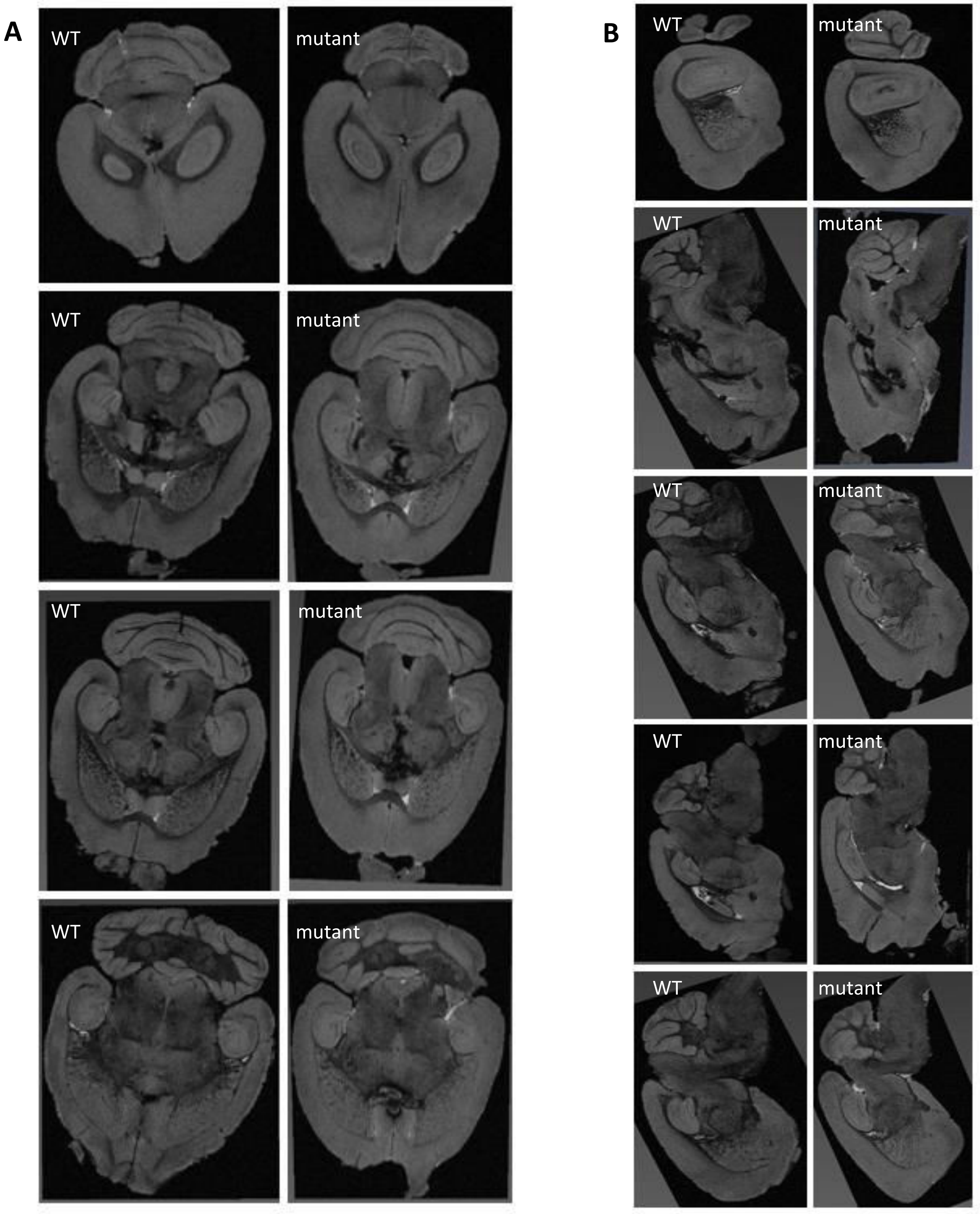

Supplementary Figure 2. **(A)** Transverse plane images from WT and mutant mouse brains after ex vivo MRI scan in a 9.4 Tesla magnet. **(B)** Sagittal images from WT and mutant mouse brains after ex vivo MRI scan in a 9.4 Tesla magnet.
